# Supplementary material for: Training convolutional neural networks with the Forward–Forward Algorithm
Source: Sci Rep. 2025 Nov 4;15:38461. doi: 10.1038/s41598-025-26235-2 (PMC12586560; doi:10.1038/s41598-025-26235-2)
Supplement: Supplementary file 1 — Supplementary Information. [file 41598_2025_26235_MOESM1_ESM.pdf]

Supplementary Materials

Appendix A: encoding the spatially-extended labels

Here, we provide more details concerning the two approaches we used to encode the proposed spatially-extended labels. We found that the two techniques have some complementary pros and drawbacks, which we discuss in the main text.

Fourier Waves labeling

Let an input image be represented as a function with spatial coordinates *(u,v)* defined over the image grid of size *HxW.*

Each pattern associated with a specific label is defined by its discrete Fourier spectrum:

$$y(k_x, k_y)$$

For a given class, *C,* we assign a specific spatial vector chosen from a predefined set of frequency–orientation pairs. The set can be manually created by the user or automatically obtained by randomly electing the parameters pairs:

$$\mathbf{k}_c = (k_x^{(c)}, k_y^{(c)})$$

Consequently, the spectrum is initialized as:

$$y(k_x, k_y) = \left(\begin{cases} A & (k_x, k_y) = \mathbf{k}_c \\ A & (k_x, k_y) = -\mathbf{k}_c \\ 0 & \text{otherwise} \end{cases} \right)$$

where *A>0* is a fixed amplitude.

By applying the inverse 2D Fourier transform, we obtain the spatial-domain label carrier:

$$\mathscr{F}^{-1}(y(u,v)) = \frac{A}{HW} e^{i(k_x^{(c)} u + k_y^{(c)} v)} + \frac{A}{HW} e^{-i(k_x^{(c)} u + k_y^{(c)} v)}$$

This simplifies to a monochromatic plane wave of orientation *θc* and spatial frequency *// **kc** //*

$$y(u,v)) = \frac{2A}{HW} cos(k_x^{(c)} u + k_y^{(c)} v) \qquad \theta_c = arctan(k_y^{(c)} / k_x^{(c)})$$

To guarantee compatibility across datasets and prevent saturation, the label pattern is rescaled to [0,1] and the final labeled image *Xc* is a superposition of the original image *X* and the label pattern:

$$x_c(u,v) = (1-K)x(u,v) + Ky_c(u,v)$$

Where K is a hyperparameter in the range [0, 1] that quantifies the contribution of the label signal relative to the original image content.

To study the MNIST, we considered two possible sets of waves, reported in Figures S1 and S2. The first set, characterized by varying frequencies and orientation of the waves, performed slightly better on MNIST dataset on a set of different FF-trained CNNs than the second set, characterized by waves with a fixed frequency and varying orientations. Detailed results are provided in Table S1. Consequently, the study of the CIFAR10 and CIFAR100 datasets was confined to the first set of labels.

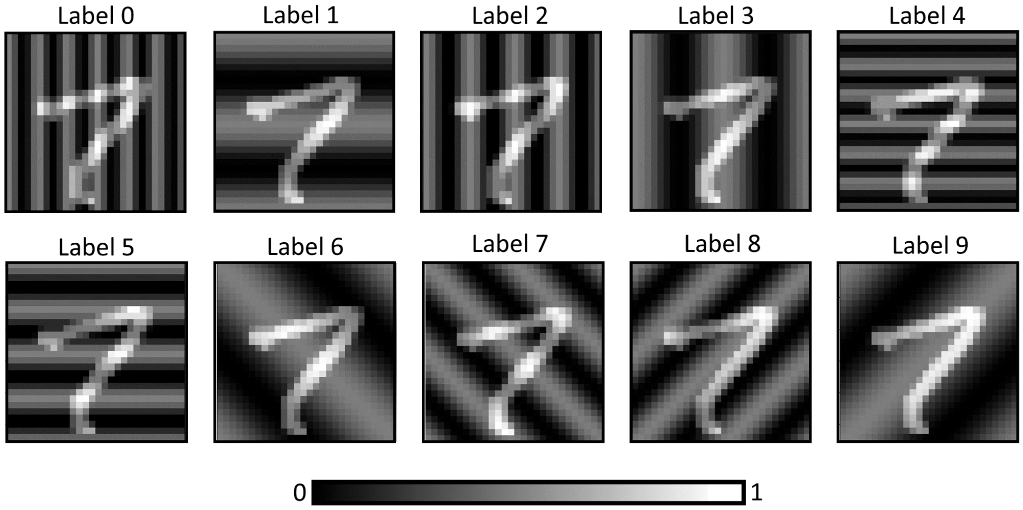

Figure S1. Label set 1 superimposed on an image of the digit 7. The 10 different labels shown here differ in both wavelengths (3 different frequencies) and orientations (0°, 45°, 90°, 135°). Only the label 7 from the MNIST data set is a part of the positive data set, while one image from the 10 images shown is randomly selected for the negative dataset.

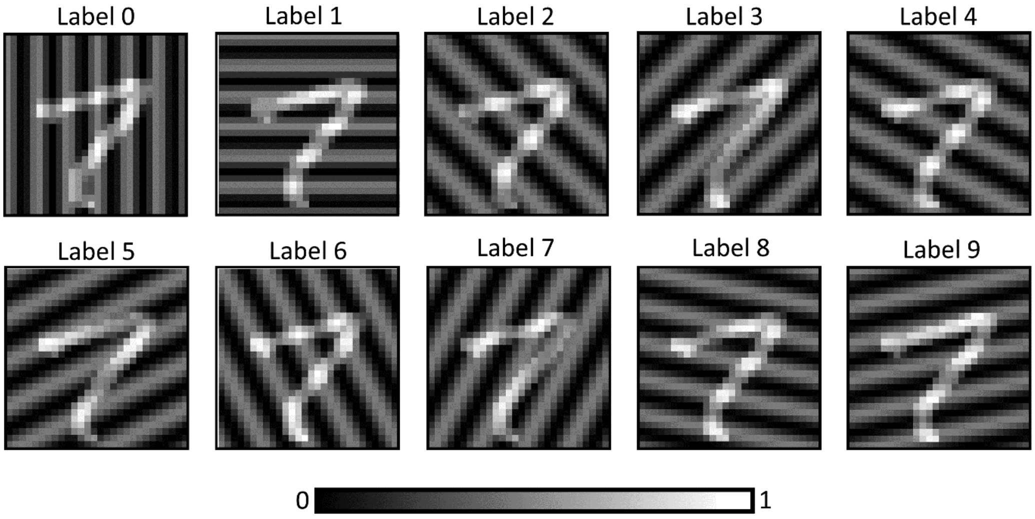

Figure S2. Label set 2 superimposed on an image of the digit 7. The 10 different labels shown here share the same wavelength and differ only in their orientation. Only the label 7 from the MNIST data set is a part of the positive data set, while one image from the 10 images shown is randomly selected for the negative dataset.

FF-CNN Network	Set 1 labels Acc. [%]	Set 2 labels Acc. [%]
32 filters of size 3x3, batch size 50	98.34 ± 0.05	**98.36 ± 0.04**
32 filters of size 7x7, batch size 25	**98.72 ± 0.02**	98.53 ± 0.05
64 filters of size 3x3, batch size 50	**98.30 ± 0.03**	98.22 ± 0.03
128 filters of size 3x3, batch size 50	**98.43 ± 0.03**	98.38 ± 0.03

Table S1. FF trained CNNs achieve higher accuracy values when labeled with set 1 instead of set 2. Four different sets of hyperparameters were trained, with the best results highlighted in bold.

Morphology-based labeling

This labeling scheme encodes class identity into low-level structural cues of the image. For example, one class may be consistently associated with a slightly blurred appearance, another with sharper contours, and another with morphological dilation. Because the transformation is blended with the original image, the semantic content remains recognizable, but the structural modification acts as an embedded label signal. Unlike symbolic encodings (e.g., Fourier overlays), morphological labeling exploits visual priors that are natural in images, making the label signal both perceptually interpretable and recognizable within convolutional networks. Moreover, differently from Fourier-based labeling, which needs to be adapted on different dataset properties of the network (e.g. convolutional filter dimensions) or of the dataset (e.g. image size), this approach is unaffected by these parameters.

Let *X* be an input image with *C* channels, height *H*, and width *W*, and *y* the classification labels for *N* classes. We define a morphological codebook as a set of class-specific operators:

$$T = \{T_0, T_1, T_2, ..., T_{N-1}\}, T_k : \Re^{C\times H\times W} \longrightarrow \Re^{C\times H\times W} \quad \text{where} \quad y \in \{0,1,2...,N-1\}$$

Each operator applies a structural or morphological modification to the image, e.g., Gaussian blurring, sharpening, edge enhancement, dilation, or erosion. The label embedding is obtained by blending the original image with its class-specific transformation:

$$x = (1-K)x + KT_y(x)$$

For CIFAR-10 we define the following set of class-specific transformations, shown in Figure S3.

Gaussian Blur (3×3) and (5x5) (labels 0 and 1), which applies a mild Gaussian smoothing with kernel size 3 or 5, reducing high-frequency noise and producing a soft or more pronounced blur. Sharpening (label 2), which convolves the image with a sharpening kernel, enhancing edges and fine details. Edge Enhancement (label 3), which extracts Sobel edges and adds them back to the image, highlighting contours. Dilation (label 4), which applies morphological dilation with a 3x3 structuring element, expanding bright regions and thickening object boundaries. Erosion (label 5) which applies morphological erosion with a 3x3 structuring element, shrinking bright regions and thinning object boundaries. Motion Blur (30° and 60°) (label 6 and 7) which simulates linear camera motion blur at a 30° and 60° angle, producing directional streaks. Blur then Sharpen (label 8), which sequentially applies Gaussian blur followed by sharpening, generating an over-smoothed yet enhanced structure. Edge then Dilate (label 9) which applies edge enhancement and then dilation, producing thicker emphasized contours.

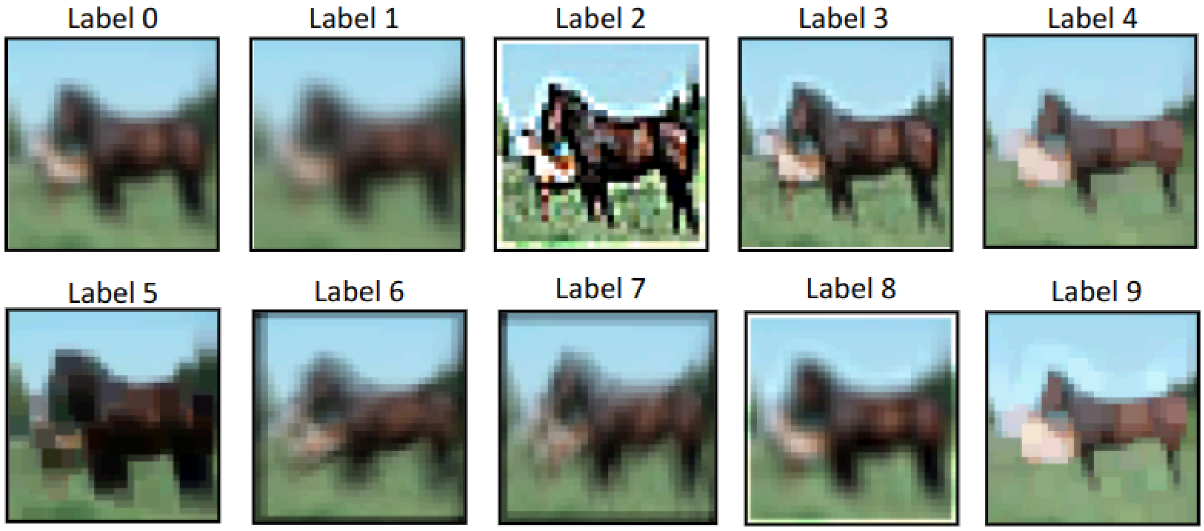

Figure S3. Morphology-based labels set applied to an image of the CIFAR10 dataset. The 10 different labels shown here have been chosen among a set of 2000 alternatives by minimizing the internal correlation among the different patterns composing the set.

Appendix B: contribution of the first layer

In order to investigate the effect of the goodness of the first convolutional layer, we train the CNN configurations reported in Figure 3 again, but this time including the first layer in the goodness computation. Figure S4, reports the results, highlighting that the training of the first layer affects the speed of convergence of the next layers. Adding the first layer also reduces the overall accuracy of the network by approximately 2%.

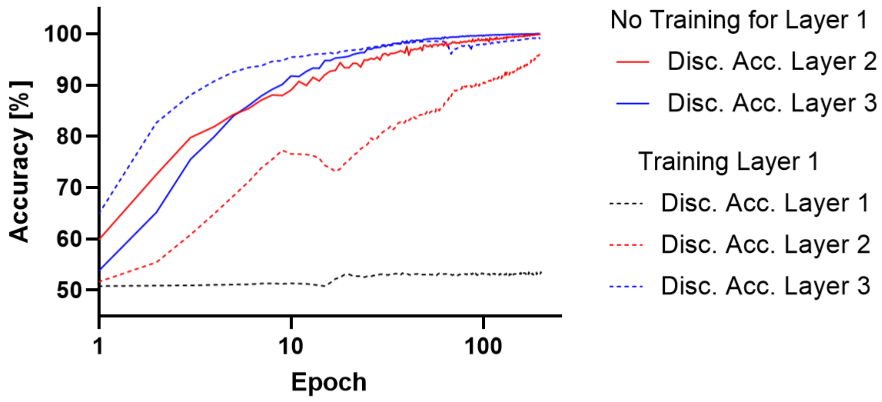

Figure S4. Our implementation of FF trained CNNs does not require the inclusion of the goodness of the first layer during training. Continuous lines represent evolution of the discrimination accuracy during the training phase, when the first layer is not included. Dashed lines represent the discrimination accuracy evolution if its goodness is included.

Appendix C: CIFAR10 and CIFAR100 CNN architecture

While a CNN of three convolutional layers is sufficient to analyze the MNIST dataset, for more complex datasets such as CIFAR10 or CIFAR100, deep networks are needed. A schematic of the convolutional section of the FF-trained CNN we used to analyze these datasets is provided in Figure S5. This network is characterized by six convolutional layers, with an increasing number of filters of 5x5 pixels. For inference, we used the linear classifier-based technique.

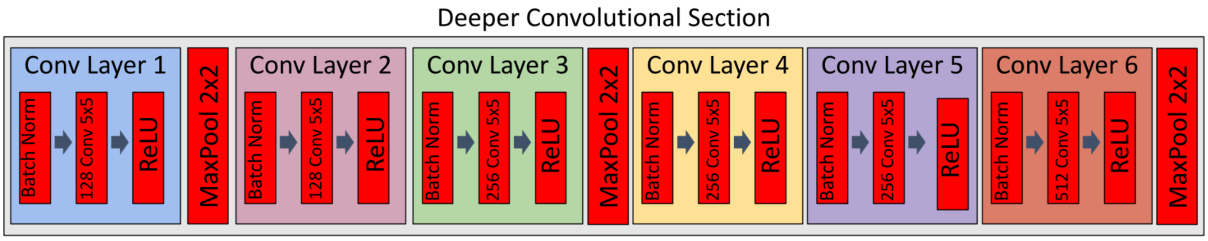

Figure S5. Convolutional section of the FF-trained CNN we used to analyze both CIFAR10 and CIFAR100 dataset. Compared to the architecture used for the MNIST dataset, we doubled the network depth by taking advantage of six convolutional layers.

Appendix D: influence of hyperparameters on MNIST dataset

This section discusses the search for optimal hyperparameters and architectural options we performed to obtain the result reported on MNIST for the optimized configuration. The performance of BP trained neural networks depends on the specific value of the hyperparameters used during training. There is no reason to expect this to be different for FF training. We limited our search to networks of three layers of convolutional filters, all with the same number of filters per layer. For the choice of an optimizer, we tested the Stochastic Gradient Descent and the Adam optimizer, with the latter consistently outperforming the former. For the learning rate of Adam optimizer, we evaluated the range between 10^{-2} and 10^{-7}. A learning rate of $5x10^{-5}$ yielded the best results and was therefore chosen as the default. As shown for the optimized configuration, we found that after training for 200 epochs, the network had converged in all configurations tested, which also made this value our default.

Filter dimensions, layer width, and batch size

Figure S6a shows that the accuracy decreases with smaller filter sizes. A possible explanation is that smaller filters have more problems in identifying the wavelengths of the label waves. For 7x7 and 5x5 filters we additionally observe an increase in accuracy for smaller batch sizes: this might be a generic feature due to the dual nature of the training data. Figure S6b confirms the increase in accuracy with the number of filters per layer for all filter sizes, which was first shown in Figure 3a, with the accuracy decreasing with smaller filter size.

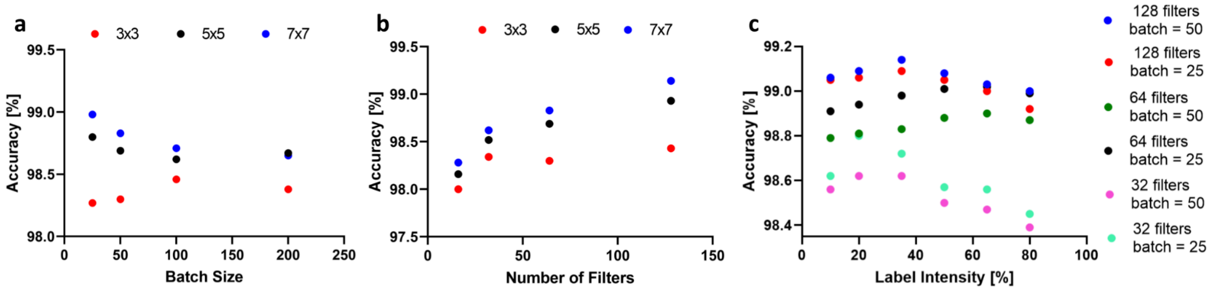

Figure S6. FF-trained CNN benefits from small batch sizes and a large number of filters, while the relative label intensity K depends on these two parameters. (a) Accuracy as a function of batch size and filter size (3x3, 5x5, or 7x7 pixels) in a network of three layers of each 64 filters. (b) Accuracy values obtained by varying the number of filters per layer (16, 32, 64, 128) and the filter size using a batch size of 50. For inference, we used the linear classifier approach. (c) Accuracy values, obtained for training with 7x7 filter size as a function of the relative label intensity K. For inference, the goodness approach was used.

Inference: linear classifier vs. goodness approach

As described in the methods section, there are two ways for making inference with a supervised FF trained neural network. Table S2 shows that the goodness computation approach beats the linear classifier over a variety of different CNN hyperparameter configurations, and this finding agrees with the proposal of Hinton [5]. However, the slightly superior results come together with a ten-times larger computational cost of the goodness comparison because each image in the dataset must be processed for each label in order to perform the classification task. Consequently, the goodness approach might only be justified for a classification task with a limited number of labels. Moreover, as shown in Figure 3a, the linear classifier approach is more robust for non-optimal configurations, making it useful during preliminary studies or characterizations.

FF-CNN Network	Linear Classifier Accuracy [%]	Goodness Accuracy [%]
128 filter of size 7x7, batch size 50	99.14 ± 0.02	$\mathbf{99.16 \pm 0.02}$
128 filter of size 5x5, batch size 50	98.93 ± 0.04	$\mathbf{99.04 \pm 0.03}$
128 filter of size 3x3, batch size 25	98.63 ± 0.03	$\mathbf{98.74 \pm 0.03}$

Table S2: Using the goodness approach yields a higher accuracy during inference than the linear classifier, albeit at 10 times higher computational cost. Three different sets of hyperparameters were trained, with the best results highlighted in bold.

Effect of the relative labeling intensity K

In studying the effect of the relative label intensity K, we focus on the hyperparameters that exhibit the highest accuracy values: 32, 64 or 128 convolutional filters per layer, filter size 7x7, and batch sizes of 25 and 50 images. The results presented in Figure S6c show that optimizing K leads to an increase in accuracy of up to 0.2%. The optimal value of K depends on the width of the layer but not on the batch size. Specifically, the 32 neurons per layer architectures achieve the highest accuracy for K approximately 20%, for 64 neurons per layer the optimal K increases to approximately 65%, for 128 neurons per layer it drops again to approximately 35%.

Appendix E: layerwise Class Activation Maps

We computed CAMs related only to each independent layer of the FF-trained CNN. Results are reported in Figure S7.

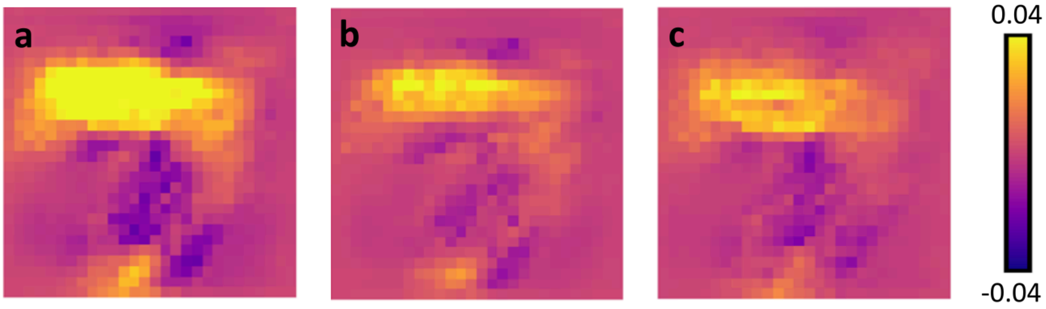

Figure S7. CAMs show that the different layers of the FF-trained CNN provide similar, but yet distinguishable information. (a) shows the CAM obtained from considering both layer 2 and layer 3 together. (b) and (c) show the CAMs obtained respectively only from layer 2 and layer 3.

Appendix F: FF performance on CIFAR10 dataset

In Table S3, we report a comparison among our results on the CIFAR10 dataset and alternative methods which extend or work on the FF algorithm. We consider both pure FF approaches and hybrid methods, which take inspiration from the FF algorithm but still perform limited backpropagation. Interestingly, the table shows that our method achieves results comparable to pure FF approaches. Only hybrid methods that incorporate limited backpropagation achieve significantly higher performance, indicating that the difference lies in the use of backpropagation rather than in the application of labels to the dataset images.

Method	Accuracy %	Details
Proposed	68.6 ± 0.5	6 layers, spatial labeling, batch normalization, multilayer inference
Trifecta [45]	65.8 ± 0.8	6 layers, batch normalization, Symba [30] loss
DeeperForward [47]	81.8 ± 0.3	14 layers, convolutional group channels, synaptic pruning, mean goodness, residual structure
FF Advance [46]	54.7	4 layers
Symba [30]	59.1	MLP, Symba loss
Trifecta [45]	75.2 ± 0.7	6 layers, batch normalization, Symba [30] loss, **OLU**
FF Advance [46]	81.2	6 layers, convolutional group channels, channel-wise loss function, **training through chunked local updates**, multilayer inference
CwC [44]	78.1 ± 0.4	Channel-wise Grouped Convolutions, **training through chunked local updates**

Table S3: FF-based networks performances on CIFAR10 dataset. Green background highlights our method, while light red and light blue boxes are associated with pure FF and hybrid methods, which also use backpropagation-based optimizations (highlighted in bold), respectively.

Appendix G: FF performance on CIFAR100 dataset

In Table S4, we report a comparison among our results on the CIFAR100 dataset and alternative methods which extend or work on the FF algorithm. At the moment, work on CIFAR100 is still very limited. We consider both pure FF approaches and hybrid methods, which take inspiration from the FF algorithm but still perform limited backpropagation.

Method	Accuracy %	Details
Proposed	38.2 ± 0.5	6 layers, 100 epochs, optimized spatial labeling via convergence analysis, batch normalization, multilayer inference
Trifecta [45]	28.7 ± 0.4	6 layers, batch normalization, Symba [30] loss, 100 epochs
Trifecta [45]	35.8 ± 0.2	6 layers, batch normalization, Symba [30] loss, 200 epochs
Trifecta [45]	35.3 ± 0.2	6 layers, batch normalization, Symba [30] loss, **OLU**
CwC [44]	51.2 ± 0.2	Channel-wise Grouped Convolutions, **training through chunked local updates**

Table S4: FF-based networks performances on CIFAR100 dataset. Green background highlights our method, while light red and light blue boxes are associated with pure FF and hybrid methods, which also use backpropagation-based optimizations (highlighted in bold), respectively.

Appendix H: minimizing redundancy in label patterns for CIFAR-100

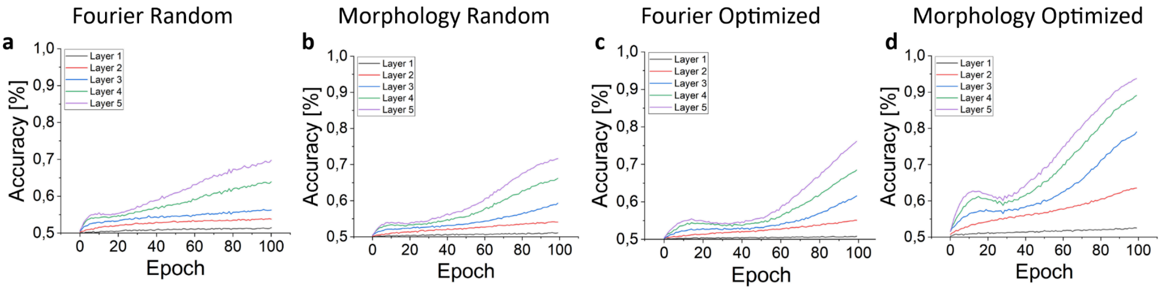

Figure S8. Minimizing correlation between labels improves the learning. (a) and (b) show the training accuracy curves for our proposed CNN for CIFAR100 dataset, trained by randomly selecting as labels 100 of the 2000 Fourier and morphology-based labels candidates, respectively. For (c) and (d), candidates were automatically selected by minimizing the internal correlation among the labels.
